# Sexual and reproductive health literacy and its associated factors among adolescents in Harar town public high schools, Harari, Ethiopia, 2023: a multicenter cross-sectional study

**DOI:** 10.3389/frph.2024.1358884

**Published:** 2024-10-15

**Authors:** Adera Debella, Aklilu Tamire, Kasahun Bogale, Bekelu Berhanu, Hanan Mohammed, Alemayehu Deressa, Mulugeta Gamachu, Magarsa Lami, Lemesa Abdisa, Tamirat Getachew, Saba Hailu, Addis Eyeberu, Helina Heluf, Henok Legesse, Ame Mehadi, Jemal Husen Dilbo, Lensa Angassa Wkuma, Abdi Birhanu

**Affiliations:** ^1^ School of Nursing and Midwifery, College of Health and Medical Sciences, Haramaya University, Harar, Ethiopia; ^2^ School of Public Health, College of Health and Medical Sciences, Haramaya University, Harar, Ethiopia; ^3^ School of Laboratory Science College of Health and Medical Science, Haramaya University, Harar, Ethiopia; ^4^ School of Medicine, College of Health and Medical Sciences, Haramaya University, Harar, Ethiopia; ^5^ Department of Public Health, Rift Valley University, Harar, Ethiopia

**Keywords:** adolescents, sexual, reproductive, health, literacy

## Abstract

**Background:**

In sub-Saharan African countries, including Ethiopia, the utilization of sexual and reproductive health information during adolescence is considered to be low. The aim of this study was to assess the level of sexual and reproductive health literacy among adolescents in Ethiopia as well as the factors associated with sexual and reproductive health literacy in this population.

**Methods:**

An institutional-based cross-sectional study design was employed. Systematic sampling methods were used to select 909 study participants. A validated scale was used, consisting of 31 questions with a 5-point Likert scale. A total score was computed, ranging from 31 (minimum score) to 155 (maximum score), which was finally categorized into limited and adequate sexual and reproductive health literacy. A multivariate linear regression model was fitted to determine the factors influencing adolescents’ sexual and reproductive health literacy.

**Results:**

The percentage of adolescents with slightly adequate and excellent sexual and reproductive health literacy was 38.9% and 6.3%, respectively. On the other hand, 677 (74.5%) participants overall had limited sexual and reproductive literacy. Healthcare workers and reading books were the preferred sources of sexual and reproductive health information that were associated with higher sexual and reproductive health literacy by 6.42 (95% CI 1.62–11.22) and 6.57 (95% CI 1.62–11.22), respectively. Adolescents' ability to pay for their healthcare was associated with better sexual and reproductive health literacy by 13.76 times (95% CI 8.21–19.32).

**Conclusion:**

More than three-quarters of the adolescents had limited sexual and reproductive health literacy. Sources of sexual and reproductive health information, including healthcare workers, books, and the Internet, were significantly associated with adolescents’ sexual and reproductive health literacy. Hence, primary stakeholders need to incorporate sexual and reproductive health into the curriculum at high schools.

## Introduction

The world is home to 1.2 billion individuals aged 10–19 years (1). The adolescent population is evolving dynamically, shaped by theoretical constructs informed through physiologic, psychosocial, temporal, and cultural views. The age range of 10–19 years marks a crucial developmental phase, typically defined as the period between the onset of puberty and the formation of social identity (2). Globally, nearly 11% of all pregnancies involve adolescents aged 15–19 years, and approximately 95% of these pregnancies occur in low- and lower-middle-income countries (3).

Sexual and reproductive health literacy (SRHL) is the understanding and application of sexual and reproductive health (SRH) information. It extends beyond health benefits and addressing SRHL issues is a top priority in developing countries. For instance, early pregnancy and marriage are more likely to occur in poor, poorly educated, and non-urban communities than in other communities (4). In developing countries, adolescents are at greater risk for unsafe abortion, child maternal death, sex-based infections, and sexually transmitted infections (STIs), including HIV/AIDS. In addition, they are more vulnerable to negative social outcomes from school absenteeism, early marriage, and poverty. These factors contribute to a cycle of absolute poverty and low literacy, thereby increasing the risk of early marriage (5).

Adolescent sexual and reproductive health is still a major public health concern in sub-Saharan Africa, especially for adolescent girls (6). In this region, adolescents constitute the highest proportion (32%) of young people aged 10–24 years (7), with a further 81.8%, 63%, and 42.5% of those adolescents attending primary, lower, and upper secondary schools, respectively (8).

There are 34.4 million youths aged 10–24 years in Ethiopia, making it the second largest population in the country. Recent evidence shows that more than 5 million Ethiopian adolescents are enrolled in high school (9). Despite these findings, a systematic study in Ethiopia showed that the utilization of sexual and reproductive services is low. Even though the government of Ethiopia gives attention to the sexual and reproductive health of adolescents (10), the prevalence of child marriage, adolescent motherhood, unplanned pregnancy, young age at first marriage, and median age at first sexual intercourse are high (11). National data from Ethiopia show that the general literacy of adolescents increased steadily between 2011 and 2016, but sexual and reproductive health literacy was not mentioned (11). Hence, the aim of this study was to assess adolescent sexual and reproductive health literacy in high schools in Harar town, Ethiopia, to gain insight into current state of sexual and reproductive health literacy and determine its relationship to factors such as sociodemographic variables, sexual characteristics, behaviors, lifestyles, and practices. The findings of this study will contribute to the design of health interventions aimed at improving the SRHL of adolescents.

## Material and methods

### Study area and period

The study was conducted in public secondary schools of Harar town, Ethiopia. According to the 2007 population projection by the Ethiopian Central Statistical Agency (CSA), the estimated total population of Harar town was 122,000. Of this, the adolescent population is estimated to be 99,368 (49,727 male adolescents, 49,641 female adolescents) (12). According to the Harar Town Education Bureau, the town has seven public secondary schools covering grades 9–12. The study was conducted in four of these schools: Aboker, Harar Senior, Shekib, and Jegnoch high schools. The study was conducted between 31 August and 19 September 2023.

### Study design and population

The study employed an institutional-based cross-sectional study design. The source population consisted of all secondary school adolescents aged 14–19 years, including both male and female adolescents. Adolescents aged younger than 14 years were excluded from the study because they were less likely to fit the study’s variables, such as sexual behavior.

### Sample size determination

The sample size was determined using a single population proportion formula, ${\left[{\left(Z_{a/2}\right)}^{2}(p)(1-p)\right]/d}^{2}$, with the following assumptions, as there was no previous related evidence: 95% confidence interval (CI), 4% margin of error (d), 5% non-response rate, and 50% of the proportion (p) of SRHL among high school adolescents. The calculated sample size was 600. Considering 1.5 for the design effect and a 5% non-response rate, the final sample size was 945.

### Sampling techniques

**Figure 1:**
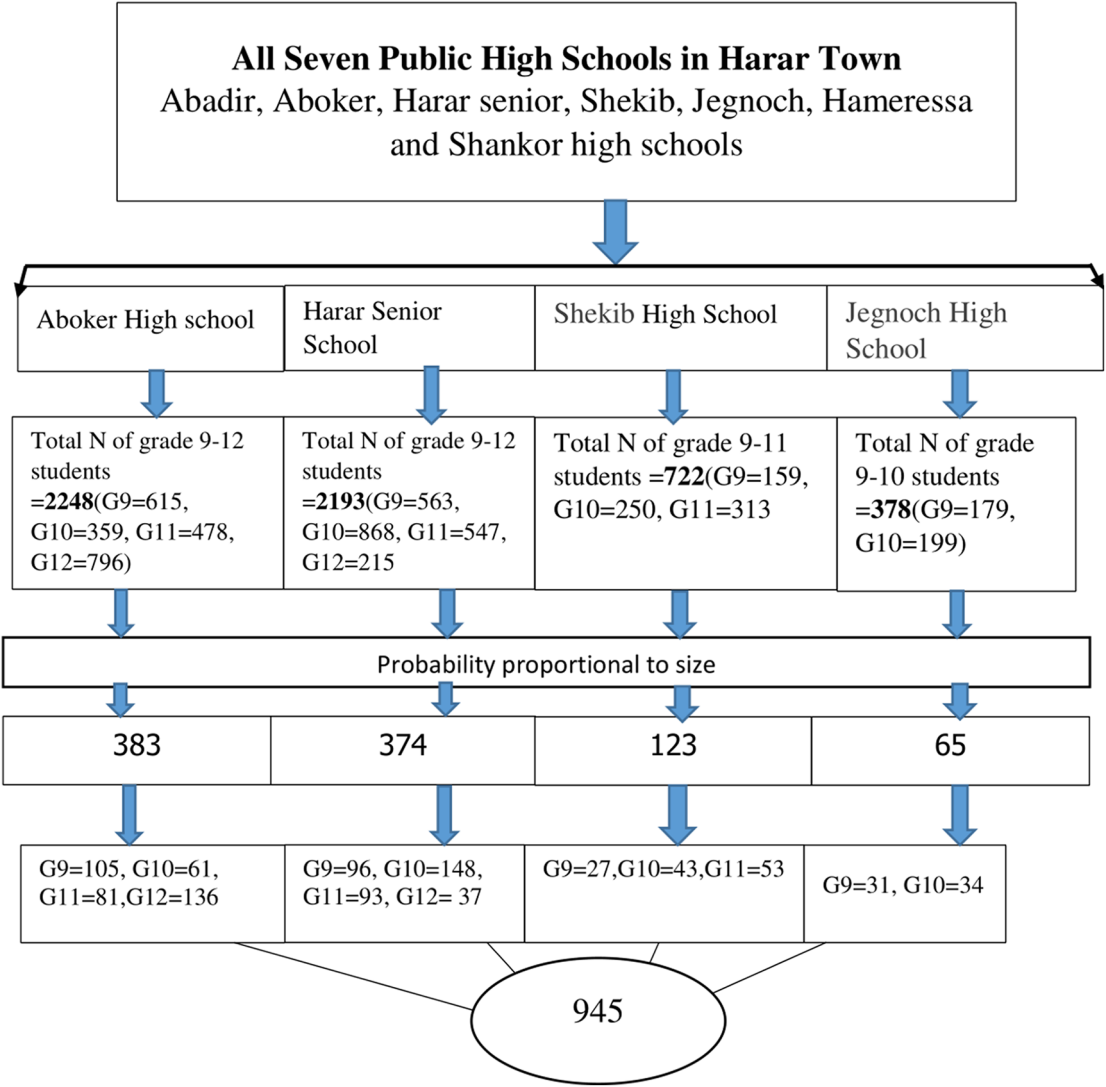
Diagrammatic representation of SRHL study participants’ selection at Harar town in 2023. Key: G9 = grade 9, G10 = grade 10, Grade 11 = grade 11, Grade 12 = grade 12. SRHL, sexual and reproductive health literacy.

Simple random sampling was used to select the high schools, whereas a multistage random sampling technique was employed to select a representative sample of students. The number of study participants from each school, grade, and section (grades 9–12) was calculated proportionally to ensure representative allocation. The number of study participants from each school was calculated proportionally using the equation nf = (no × n1)/N, where nf is the number of participants from each school, no is the calculated sample size, n1 is the total number of students in each school, and *N* is the total number of students from the four (sampled) schools. On the other hand, the number of participants from each grade (section) was calculated using the equation mf = (mo × m1)/M, where mf is the number of study participants from each grade (section) (grades 9–12), mo is the number of study participants from each school, m1 is the total number of students in each grade, and mf is the total number of students in each school. Then, the required number of study participants was selected using simple random sampling from each section, with the sample size allocated to each section determined by Probability Proportional to Size (PPS). Hence, after the study participants were allocated to each section using PPS, simple random sampling methods were applied to recruit the required number of participants from each school and section (Figure 1). Of 945 students who were requested to bring their guardians’ signed informed consent, 8 students did not. A discussion with the remaining 937 students was held regarding the overall study and voluntary participation. Students were given the opportunity to raise any issues during the discussion and were also invited to questions before, during, and after data collection. After the discussion, data collection from the 937 students began immediately. Of the students, 909 completed the questionnaire while 28 returned incomplete questionnaires and were excluded from the analysis.

### Data collection tools and techniques

Pretested, validated, self-administered, and structured questionnaires were used to collect the data. The sexual characteristics and literacy tools were adapted from previously published literature and contextualized to local realities (13, 14). The tools were closed, open-ended, and had three parts: sociodemographic characteristics, sexual and reproductive characteristics, and SRHL. The SRHL part has 31 questions containing a 5-point Likert scale (very difficult, difficult, medium, easy, and very easy), with minimum and maximum scores of 31 and 155 points, respectively. It contains accessibility and ability to understand and identify appropriate reproductive health information concepts. In addition, it concerned reproductive health, including sexually transmitted infection, unwanted pregnancy, and family planning. The Likert scales were in the range of 0–4. Each question had a scoring scale reflecting the ability to access, understand, appraise, and apply SRH information from the scale “very difficult” = 0 to “very easy” = 4. The questionnaire was originally developed in English, translated into local languages (Afan Oromo and Amharic), and then translated back into English by language experts to check for language consistency and accuracy. The Amharic and Afan Oromo questionnaires were then used to collect the data. Participation was fully voluntary and oral consent was obtained from the participants as well as written informed consent from their guardians.

### Study variables

In this study, in addition to the Likert scale, both categorical and continuous variables were used. All 31 SRHL questions were based on the Likert scale while age was the only continuous variable. On the other hand, the following categorical variables were used: residence, religion, language, educational level, with whom the adolescents were living, parents’ occupation, access to Internet, sources of SRH, attending SRH at school, ever used SRH services, ever counseled on SRH, ever learnt about puberty, ever experienced sexual intercourse, ever used contraceptives, know the cause of cervical cancer, know the availability of the HPV vaccine, know where HIV/AIDS tests can be offered, ever get tested for HIV/AIDS, and know where to receive SRH service. All types of variables were collected.

The dependent variable was reproductive health literacy, which was computed from 31 SRHL questions, whereas social demographic characteristics, including age, sex, marital status, source of reproductive health information (Internet, media use, family and peer influences, mass media, education level), and health status of the adolescents (including current health status, doing physical exercise, having disability, having chronic illness, affordability for healthcare, and alcohol consumption) were independent variables.

### Data quality control

A validated structured data collection tool was used to collect the data. The questionnaire was originally developed in English. It was then translated into local languages and subsequently translated back into English to check the appropriateness of translation. The Amharic questionnaire (translated from English) was used to collect the data. Four data collectors and two supervisors were allocated. Two days of training were given to the data collectors and supervisors on the objectives of the study, the contents of the data collection tools and procedures, and the participants’ autonomy. The questionnaires were pretested on 5% of the sample in Abadir High School before the actual data collection period to check the reliability and validity of the data collection tools, and question sequences were amended before the final data collection. The high school used for the pretest was excluded from the present study. If there was any ambiguity, the data collectors provided clarification during the data collection. The data collectors, supervisors, and principal investigators reviewed and checked the completed questionnaires for completeness, accuracy, and consistency on a daily basis. From the calculated sample size, 36 questionnaires were incomplete and excluded from the analysis.

### Data processing and statistical analysis

The collected data were entered into Epi-Data version 4.6.02 and then exported to SPSS version 25 (IBM Corp.). Cronbach's alpha was calculated as 0.965, indicating the excellent internal consistency of the questionnaire. The data cleaning, completeness, and outer layers were checked. A descriptive statistical analysis was performed, and the outputs were summarized as proportions and frequency, whereas the literacy levels were determined from a total score that was standardized from 100. Scores in the range of 0–50 were considered inadequate SRHL, 50.1–66 slightly adequate SRHL, 66.1–84 adequate SRHL, and 84.1–100 excellent SRH. Inadequate and slightly adequate were considered limited SRHL, while adequate and excellent were considered optimum SRHL. Literacy status was then determined by two categories: limited and optimal SRH. In addition, once the relationship between independent and dependent variables were established, bivariate analysis or simple linear regression analysis was performed. Variables with a *p*-value >0.25 in simple linear regression were added to the multivariate linear regression model. Significance was determined at a maximum *p*-value of 0.05 and a 95% CI.

## Results

### Sociodemographic characteristics

In this study, 412 (45.3%) boys and 497 (54.7%) girls responded to the questionnaires, giving a response rate of 96%. The remaining questionnaires were excluded from the analysis because they were incomplete. The mean ± SD age of the participants was 16.8 ± 0.04 years. A total of 548 (60.3%) respondents were aged 15–17 years and 60% of the adolescents were Muslim. Regarding educational status, 390 (42.9%) were in the ninth grade. Of the adolescents, 81% reported that they lived with their parents. Moreover, nearly half of respondents (430, 47.3%) had a family member working in health-related areas. Internet access was available for 625 (68.8%) adolescents (Table 1).

**Table 1:** Demographic characteristics of school-aged adolescents in Harar town, Ethiopia, 2023 (*N* = 909).

Variables	Categories	Frequency	Percentage
Age	15–14	26	2.9
	16–17	548	60.3
	18–19	335	36.9
Sex	Male	412	45.3
	Female	497	54.7
Residence	Urban	650	71.5
	Rural	259	28.5
Religion	Muslim	551	60.6
	Orthodox	298	32.8
	Protestant	46	5.1
	Others	14	1.5
Which language do you speak most of the time?	Affan Oromo	445	49
	Amaharic	417	45.9
	Adere	24	2.6
	Somalie	8	0.9
	Other	15	1.7
Which grade are you attending now?	Grade 9	390	42.9
	Grade 10	271	29.8
	Grade 11 and 12	248	27.3
Are you living with parents?	Yes	745	82
	No	164	18
Where did live before the age of seven?	Big city	178	19.6
	Medium and small city	223	24.5
	Town	286	31.5
	Country side	182	20
	Rural	40	4.4
Do your family member works in health-related area?	Yes	430	47.3
	No	479	52.7
Do you have an internet access?	Yes	625	68.8
	No	284	31.2

### Sexual and reproductive health awareness and use among adolescents

Table 2 shows the results for sexual and reproductive health among adolescents and related knowledge and behaviors. Most adolescents (31%) preferred to receive SRH information from health professionals. Of the adolescents, 64% reported attending school-based SRH education, and one-quarter of the adolescents thought that there should be additional SRH classes. Only 20% of the adolescents reported utilizing SRH services throughout their life. Among 909 adolescents, 370 (40%) had received counseling for SRH issues. Regarding sexual activity, 128 (14.08%) adolescents had prior sexual intercourse and 98 (10.8%) had used contraception.

**Table 2:** Sexual and reproductive health characteristics among adolescents in Harar town, Ethiopia, 2023 (*N* = 909).

Variables	Categories	Frequency	Percentage
What are your sources of information about SRH issues?	School teacher	213	23.4
	Parents	101	11.1
	Friends	83	9.1
	Health professionals	290	31.9
	books/magazine	160	17.6
	Internet	34	3.7
	Others	28	3.1
Have you ever attended school-based SRH Education?	Yes	582	64.0
	No	327	36.0
How often do you think the SRH class should be given?	More	564	62.1
	Less	162	17.8
	About right	183	20.1
How often do you attend SRH classes?	Regularly	685	75.4
	Rarely	224	24.6
Have you ever utilized SRH service?	Yes	183	20.1
	No	726	79.9
Have you ever counseled for SRH issues?	Yes	370	40.7
	No	539	59.3
From where did you learn about puberty?	Teacher	764	84.1
	Friend	66	7.3
	Social media	52	5.7
	Others	27	3.0
Ever engaged in SRH education activities/SRH clubs	Regularly	246	27.1
	Rarely	663	72.9
Have you ever had sexual intercourse till now?	Yes	128	14.1
	No	781	85.9
Have you ever used contraceptives?	Yes	98	10.8
	No	811	89.2

SRH, sexual and reproductive health.

Nearly half of the school-aged adolescents (420, 46.2%) did not know about the causes of cervical cancer and 497 (45.3%) did not know about vaccines against HPV. Only 262 (28.8%) adolescents had ever been tested for HIV. Of the adolescents, 60% agreed that first sex can lead to pregnancy. A total of 408 (45%) adolescents responded that having sex halfway between periods was most likely to lead to pregnancy. More than half the adolescents (531, 58.4%) responded that using a condom was not safe during sexual intercourse. The majority of the respondents (568, 62.5%) were aware of where to find SRH information (Table 3).

**Table 3:** Sexual and reproductive awareness of adolescents with Harar town ethnicity, Ethiopia, 2023 (*N* = 909).

Variables	Categories	Frequency	Percentage
Do you know what causes cervical cancer?	Yes	489	53.8
	No	420	46.2
There is vaccine against HPV!	Yes	412	45.3
	No	497	54.7
Do you know where to take HIV test	Yes	535	58.9
	No	374	41.1
Have you ever tested for HIV/AIDS?	Yes	262	28.8
	No	647	71.2
First sex can lead to pregnancy	Yes	551	60.7
	No	358	39.4
Having sex half way between periods most likely to leads to pregnancy	Yes	408	45
	No	501	55.1
Condom use is the safest during sex	Yes	378	41.6
	No	531	58.4
Know a place where to receive SRH information	Yes	568	62.5
	No	341	37.5

HPV, human papilloma virus; HIV, human immunodeficiency virus.

### General health characteristics

Approximately one-third (288, 31.7%) of the adolescents perceived their health status as worse than the majority when they compared it with those their peers. Within the past 6 months, 277 (30.5%) respondents had faced health problems. The majority of the adolescents (391, 43%) had not done any form of physical exercise >30 min, and 81% had not consumed alcohol within the past 12 months. Of the adolescents, 8% and 7% were disabled and had a chronic disease, respectively. The ability to pay for their own healthcare was found to be very difficult for 251 (27.6%) adolescents (Table 4).

**Table 4:** General health among adolescents in Harar town, Ethiopia, 2023 (*N* = 909).

Variables	Categories	Frequency	Percentage
What is your health status as compared with people of your age?	Worse than majority	288	31.7
	Worse than few	92	10.1
	Average	198	21.8
	Better than few	170	18.7
	Better than majority	161	7.7
Have you faced health problems in the last 6 months?	Yes	277	30.5
	No	632	69.5
Do you do physical exercise >30 min?	Not at all	391	43.0
	1–2 times/month	211	23.2
	1–2 times/week	115	12.7
	Almost everyday	92	21.1
Do you have disability?	Yes	75	8.3
	No	834	91.7
Do you have a chronic illness?	Yes	64	7.1
	No	845	92.9
Do you afford for your general healthcare?	Very difficult	251	27.6
	Fairly difficult	222	24.4
	Fairly easy	298	32.8
	Very easy	138	15.2
Di you drink alcohol in the past 12 months?	Never drank	737	81.1
	<Once a month	81	9.0
	Once a month	37	4.1
	Once a week	26	2.9
	>Once a week	28	3.1

**Figure 2:**
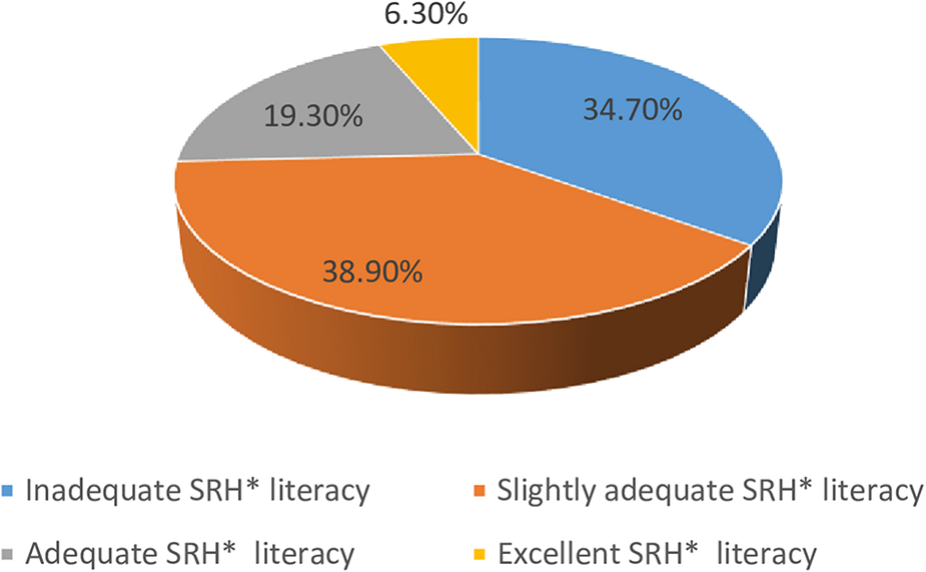
Adolescent SRH literacy classification at public school, Harar, Ethiopia. SRH, sexual and reproductive health.

The mean SRHL score was 55.7 ± 0.58, whereas the minimum and maximum SRH literacy scores were 19.4 and 96.8, respectively. Among the four measurements of SRHL, 362 (38.9%) participants had slightly adequate literacy, whereas only 57 (6.3%) had excellent SRHL (Figure 2). According to the final SRH measurements, 677 (74.5%) participants had limited SRHL and only 25% had optimal SRHL (Table 5).

**Table 5:** Final judgment of adolescents’ SRH literacy at public school in Harar town, 2023.

Overall judgment	Frequency	Percent
Limited SRH literacy	677	74.5
Optimum SRH literacy	232	25.5

SRH, sexual and reproductive health.

### Factors associated with adolescent sexual and reproductive health literacy

Multiple linear regression was fit after the following assumptions had been checked: multicollinearity (variance inflection factor < 3), autocorrelation (Watson Durbin test = 1.99), linearity (indicated by a scatter plot), and normality (indicated by a histogram). Accordingly, with healthcare workers being the preferred sources of SRH information, SRHL increased by 6.42 (range 1.61–11.22), while if the source of SRH information was books then SRHL would increase by 6.57 (range 0.85–12.29). However, if the Internet was the source of SRH information, SRHL would decrease by 10.26 (range −20.01 to −0.5). On the other hand, if adolescents could easily pay for their healthcare, their SRHL level would increase 13.76 times (range 8.21–19.32) (Table 6).

**Table 6:** Factors associated with literacy among adolescents in Harar town, Ethiopia, 2023.

Variables	Responses	*β*	*p*-value	95% CI	
				Lower limit	Upper limit
Gender	Female	2.77	0.137	−0.88	6.42
	Male	1	1		1
Age		−0.38	0.57	−1.69	0.93
Residence	Rural	−3.34	0.111	−7.45	0.76
	Urban	1	1	1	
	Yes	1	1	1	1
Internet access	No	−2.52	0.202	−6.38	1.35
	Yes	1	1	1	1
Family work in healthcare facilities	Yes	2.35	0.212	−1.34	6.04
	No	1	1	1	1
Preferred SRH information	Teacher	1	1	1	1
	Parents	0.72	0.824	−5.64	7.09
	Friends	0.42	0.904	−6.36	7.20
	Healthcare worker**	6.42	0.009	1.61	11.22
	Books/magazine*	6.57	0.024	0.85	12.29
Attend SRH class	Yes	1	1	1	1
	No	1.89	0.331	−1.92	5.70
Used SRH services	No	2.40	0.303	−2.17	7.00
	Yes	1	1	1	1
From whom you learn about puberty	Teacher	1	1	1	1
	Friend	−6.34	0.07	−13.20	0.52
	Media/television	2.44	0.529	−5.160	10.03
How often attend SRH class	Regularly	1		1	1
	Rarely	4.71	0.036	0.31	9.12
How often did you join SRH activities	Regularly	1	1	1	1
	Rarely	3.24	0.137	−1.03	7.50
Use contraceptive	No	−1.95	0.509	−7.75	3.84
	Yes	11	1	1	1
Affordability for general health	Very difficult	1		1	1
	Difficult	0.41	0.868	−4.45	5.27
	Fairly**	6.44	0.007	1.74	11.15
	Very easy**	13.76	0.000	8.21	19.32
Have you have been counseled on SRH	Yes	1	1	1	1
	No**	−3.66	0.05	−7.32	−0.00
I know from where to get SRH information	Yes				
	No**	−6.44	0.001	−10.09	−2.79
Constant		88.82	0.000	59.72	117.92

*Β*, beta coefficient compares the strength of the effect of each individual independent variable to the dependent variable (sexual and reproductive health literacy).

*Significant at *p* <0.01; **Significant at *p* <0.05.

## Discussion

This study demonstrated that more than three-quarters of the adolescents had limited SRHL. On the other hand, nearly 4 in 10 high school adolescents had slight SRHL. In the present study, the magnitude of adolescents’ SRHL was lower than that in a study from Iran, which showed that approximately 85% of adolescents had limited SRHL. This inconsistency may be due to the sample size and socioeconomic differences between the study settings (15). A limited SRHL indicates a poor level of knowledge, and poor personal skills and self-confidence in making decisions about SRH, particularly in the prevention of sexual risk behaviors (16). Adolescents’ limited SRH also leads to child marriage, unsafe abortion, increased cesarean section, social deprivation, school absence, and susceptibility to sexually transmitted diseases, including HIV/AIDS (5, 17). The following sources of SRH information were significantly associated with adolescents’ SRHL: healthcare workers, books, Internet access, ability to pay for their healthcare (fairly and very easily), getting counseling for SRH, and knowing where they could obtain knowledge about SRH. Accordingly, the magnitude of SRHL was relatively greater for students who obtained information-related SRH from healthcare workers. There is additional evidence that adolescents who obtain information from healthcare workers have good knowledge and positive attitudes toward sexual and reproductive health (15). SRH information and need-based counseling/intervention by healthcare workers are effective strategies for improving the SRHL of adolescents (18). SRH information from books/magazine was also significantly associated with SRHL status. This finding is similar to other results from a study conducted in in Iran, which revealed that adolescents who read SRH-related books and magazines were more likely to be SRH literate. Book reading contributed to more adolescents’ SRHL and also has a survival advantage, which is significantly more than reading newspapers or magazines. Book readers experienced a 20% reduction in the risk of mortality from sexually transmitted disease compared with non-book readers. Adolescents can also benefit from reading books, as they contribute to a longer life expectancy (19).

On the other hand, receiving SRH information from the Internet was directly associated with the level of SRHL. This finding is different from that of other studies (20), which may be due to the study setting and socioeconomic differences in the study areas. Currently, many studies have shown that approximately one in four adolescents uses the Internet to find SRH information. There are also websites intended to provide factual and real SRH information, which have a range of sexual health matters and virtual forums for adolescents to ask questions and participate in SRH discussions that significantly contribute to ongoing SRHL (15, 21). The current finding is similar to that of a study from another part of the world, in which black adolescents had lower SRHL and faced difficulties in paying for their healthcare (22), which may be because black adolescents have similar socioeconomic characteristics. On the other hand, a study from Southeast Asia indicated that there was no significant association between ability to pay for their healthcare and SRHL. These inconsistencies may be due to differences in the general literacy of the total population. If adults are able to pay for their healthcare more easily, this will not only help the current SRH health status but also contribute to reducing SRH complications during adulthood. Hence, the ability to pay for their healthcare has a positive impact on adolescent SRHL (22, 23).

SRH counseling is positively associated with SRHL, which is consistent with the findings of other studies (24). This may be attributed to the fact that counseling not only has positive outcomes but is also directly related to other positive outcomes from healthcare services, such as HIV-focused couples counseling increasing condom usage and reducing unprotected sexual intercourse. On the other hand, counseling also contributes to a reduction in the incidence of HIV among HIV-discordant partners. A more recent review of HIV couples also noted that effective results can arise from married and unmarried adolescent counseling. In addition, adolescent counseling is related to increased contraceptive use (25). Hence, adolescent counseling plays a significant role in contributing to adolescent SRHL and wellbeing, and online counseling provides additional protection to youth and adolescents, especially regarding SRH, mental, and psychosocial health, by increasing the number of counselors (24). This may contribute to increased sexual and reproductive health literacy. The results of this study show that students who were unaware about the source of SRH information/knowledge had lower SRHL. This is consistent with the findings of similar studies (26).

## Conclusion

More than three-quarters of the adolescents had limited SRHL. The healthcare workers, books/magazines, and the Internet were found to be significantly associated with adolescents’ SRHL. On the other hand, school-age adults who were aware of the sources of SRH information and who received SRH counseling were associated with SRHL. Furthermore, adolescents able to pay for their own healthcare was associated with SRHL. Hence, the Harari Health Bureau and other stakeholders need to add SRH sessions to schools and promote SRH through school mini media and SRH clubs.

### Operational definitions

SRH literacy: 0–50 = inadequate, 50.1–66 = slightly adequate, 66.1–84 = adequate, and 84.1–100 excellent. Limited SRH = the combination of inadequate and slightly adequate (0–66). Optimal SRH = the combination of adequate and excellent SRH literacy (66.1–100) (15).

### Operational definition

Sexual health literacy: the ability to understand and apply information regarding sexual health, decreasing the risk of STIs, and providing various benefits beyond health.

## Data Availability

The raw data supporting the conclusions of this article will be made available by the authors, without undue reservation.
